# RET/PTC Rearrangements Are Associated with Elevated Postoperative TSH Levels and Multifocal Lesions in Papillary Thyroid Cancer without Concomitant Thyroid Benign Disease

**DOI:** 10.1371/journal.pone.0165596

**Published:** 2016-11-01

**Authors:** Xuan Su, Caiyun He, Jiangjun Ma, Tao Tang, Xiao Zhang, Zulu Ye, Yakang Long, Qiong Shao, Jianyong Shao, Ankui Yang

**Affiliations:** 1 Department of Head and Neck, Sun Yat-sen University Cancer Center; State Key Laboratory of Oncology in South China; Collaborative Innovation Center for Cancer Medicine, Guangzhou, China; 2 Department of Molecular Diagnostics, Sun Yat-Sen University Cancer Center, State Key Laboratory of Oncology in South China; Collaborative Innovation Center for Cancer Medicine, Guangzhou, China; Beth Israel Deaconess Medical Center, UNITED STATES

## Abstract

RET/PTC rearrangements, resulting in aberrant activity of the RET protein tyrosine kinase receptor, occur exclusively in papillary thyroid cancer (PTC). In this study, we examined the association between RET/PTC rearrangements and thyroid hormone homeostasis, and explored whether concomitant diseases such as nodular goiter and Hashimoto's thyroiditis influenced this association. A total of 114 patients diagnosed with PTC were enrolled in this study. Thyroid hormone levels, clinicopathological parameters and lifestyle were obtained through medical records and surgical pathology reports. RET/PTC rearrangements were detected using TaqMan RT-PCR and validated by direct sequencing. No RET/PTC rearrangements were detected in benign thyroid tissues. RET/PTC rearrangements were detected in 23.68% (27/114) of PTC tissues. No association between thyroid function, clinicopathological parameters and lifestyle was observed either in total thyroid cancer patients or the subgroup of patients with concomitant disease. In the subgroup of PTC patients without concomitant disease, RET/PTC rearrangement was associated with multifocal cancer (P = 0.018). RET/PTC rearrangement was also correlated with higher TSH levels at one month post-surgery (P = 0.037). Based on likelihood-ratio regression analysis, the RET/PTC-positive PTC cases showed an increased risk of multifocal cancers in the thyroid gland (OR = 5.57, 95% CI, 1.39–22.33). Our findings suggest that concomitant diseases such as nodular goiter and Hashimoto's thyroiditis in PTC may be a confounding factor when examining the effects of RET/PTC rearrangements. Excluding the potential effect of this confounding factor showed that RET/PTC may confer an increased risk for the development of multifocal cancers in the thyroid gland. Aberrantly increased post-operative levels of TSH were also associated with RET/PTC rearrangement. Together, our data provides useful information for the treatment of papillary thyroid cancer.

## Introduction

Papillary thyroid carcinoma (PTC) is the most common malignancy of the thyroid gland. Rearrangement of the RET proto-oncogene resulting in upregulated RET protein tyrosine kinase receptor activity is believed to play a causative role in PTC pathogenesis [1]. RET/PTC1 (fusion of RET with H4) and RET/PTC3 (fusion RET with ELE1) are the most prevalent variants of RET/PTC [2]. Recent reports have shown that RET/PTC rearrangement is uniquely associated with PTC. Studies on the effect of this oncogene on thyroid hormone homeostasis would provide a deeper understanding of PTC. Moreover, it remains unclear whether concomitant thyroid benign diseases such as nodular goiter and Hashimoto's thyroiditis influence the relationship between RET/PTC rearrangements and PTC.

Patients presenting with thyroid nodules are evaluated for levels of serum thyroid stimulating hormone (TSH), free triiodothyronine (fT3) and thyroxine (fT4), thyroglobulin (TG), and anti-thyroid peroxidase antibody (ATPO) [3, 4]. These factors determine the balance of thyroid hormone homeostasis. The 2015 American Thyroid Association (ATA) management guidelines report that higher serum TSH levels, even those within the upper part of the reference range, were associated with an increased risk of malignancy in thyroid nodules, as well as more advanced stage thyroid cancer [5, 6]. To date, however, a systematic study of these thyroid specific parameters in the context of papillary carcinomas with RET/PTC has not been performed.

In this study, we performed detailed morphologic assessment of PTC and measured thyroid hormone levels prior to and following surgery. Furthermore, we analyzed the correlation between RET/PTC rearrangement and microscopic features, clinical manifestations and thyroid function parameters in thyroid cancer with or without thyroid inflammatory diseases.

## Material and Methods

### Participants and tumor samples

This protocol was approved by the Ethics Committee of Sun Yat-sen University Cancer Center, Guangdong, China. Written informed consent was obtained from all patients at their first visit.

RET/PTC rearrangement was examined in a total of 114 PTC cases enrolled at the Sun Yat-sen University Cancer Center, Guangdong, China between 2011 and 2013. All subjects were unrelated Chinese Han inhabiting southern China. Individuals who had undergone thyroidectomy or had a history of other malignant neoplasms were excluded. Matched cancerous and non-cancerous specimens were obtained from study participants when they underwent surgery. The tissues were excised and immediately frozen in liquid nitrogen and stored at -80°C within 30 min. Study participants included 29 women (25.4%) and 85 men (74.6%), ranging in age from 13 to 76 years (mean age, 41 years).

### Data collection

Medical records and surgical pathology reports were reviewed to obtain demographic parameters and pathologic characteristics of the tumors. Slides were independently reviewed by two pathologists to confirm the diagnosis of PTC and concomitant diseases such as nodular goiter and Hashimoto's thyroiditis. Information of primary tumor size, stage grouping, extrathyroid extension and metastasis were assessed based on the National Comprehensive Cancer Network (NCCN Guidelines, Version 2, 2014) on thyroid cancer recommendations (https://www.nccn.org/). Primary and secondary pathological changes were examined and described in detail. The evaluation of thyroid function was performed by the clinical laboratory at the Sun Yat-sen University Cancer Center. Serum TSH, fT3, fT4, TG and ATPO levels were measured before surgery and one month after surgery.

### Detection of RET/PTC rearrangements

**Fig 1:**
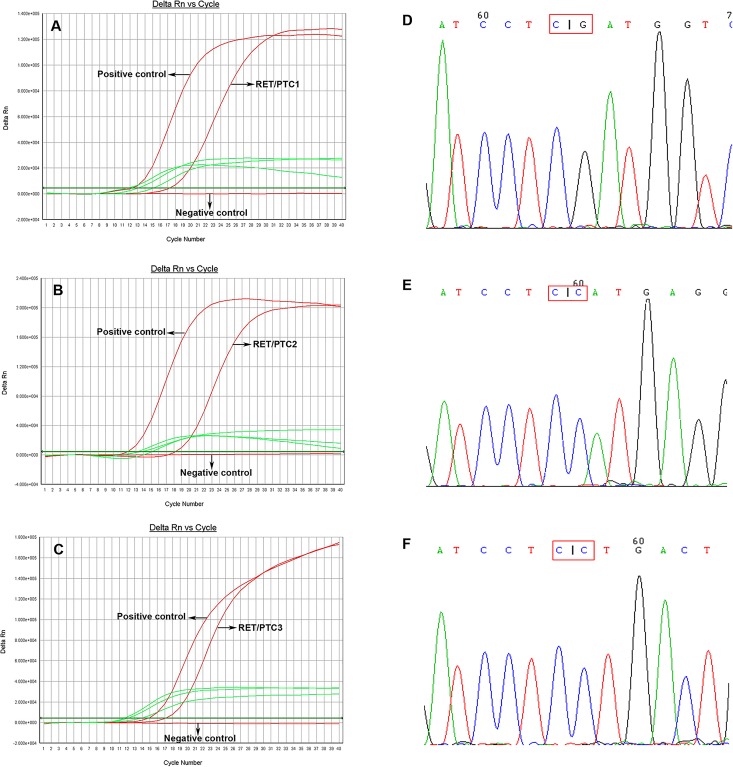
Amplification and sequencing of RET/PTC variants. The qRT-PCR amplification curves show amplification of RET/PTC1, 2 and 3 (red curves) and GAPDH control (green curve). (A) Tumor with the RET/PTC1 fusion gene. (B) Tumor without the RET/PTC2 fusion gene. (C) Tumor without the RET/PTC3 fusion gene (D) DNA sequencing analysis of PCR products containing RET/PTC1. (E) DNA sequencing analysis of PCR products containing RET/PTC2. (F) DNA sequencing analysis of PCR products containing RET/PTC3.

RET/PTC 1, 2 and 3 rearrangements were detected using TaqMan RT-PCR. Primers and probes used in this experiment were designed by Applied Biosystems (Thermo Fisher Scientific). Amplification and analysis was performed on an ABI 7500 Real Time PCR System (Applied Biosystems, CA, USA). The assay was performed with primers designed to flank the fusion point between RET and its partner genes. The sequences of primers and probes are summarized in S1 Table. The three variants of RET/PTC were detected by multiplex PCR using the following cycling parameters (Fig 1): 95°C for 2 min; 95°C for 25 s, 65°C for 20 s × 20 cycles; 95°C for 25 s, 62°C for 20 s × 40 cycles. For each PCR reaction, all controls were performed in parallel. Plasmids containing the targeted fusion sequences were used as positive controls. Negative controls had no RET/PTC rearrangements. Each rearrangement detected by RT-PCR was further confirmed by direct Sanger sequencing (Fig 1).

### Statistical analysis

All statistical analyses were performed with SPSS 16.0 software (SPSS, Chicago, IL, USA). The distribution of qualitative variable was examined using a χ^2^ test or Fisher’s exact test. The distribution of quantitative variable was examined using the Kolmogorov–Smirnov test. The variables that did not follow a normal distribution were compared between two groups by the Mann–Whitney U test. The corresponding variables are shown as the medians (with 25–75% quartiles). Primary tumor size and changes in thyroid function parameters before and after surgery that conformed to a normal distribution were compared between two groups using a Student’s t-test and the means were shown (with standard deviation). Multifactorial logistic regression analysis using likelihood-ratio test was performed to assess the association strength between RET/PTC and multifocal cancer in the thyroid gland and to explore the factors that associated with concomitant diseases in PTC adjusting for demographic parameters and pathologic characteristics. A two-sided P value of less than 0.05 was considered statistically significant.

## Results

### RET/PTC rearrangement

No RET/PTC1, 2, or 3 rearrangements were detected in the 114 non-cancerous tissues, indicating that RET/PTC rearrangements occurred exclusively in PTC. In the 114 matched cancerous tissue samples, rearrangements were identified in 27 cases, giving an overall prevalence of 23.68%. Of the 27 positive cases, 92.6% (25/27) of rearrangements were RET/PTC1 or RET/PTC3. Therefore, subsequent analysis focused on these two variants.

### Main effect of RET/PTC rearrangements on clinicopatholoigic characteristics, lifestyle and thyroid function in all thyroid cancer patients

In our initial analysis, we disregarded the potential confounding effects of common concomitant diseases such as nodular goiter and Hashimoto's thyroiditis for our analysis of 114 PTC cases. We did not observe any statistically significant association between RET/PTC rearrangements and clinicopathologic features including primary tumor size, T stage, N stage, stage grouping, number of lesions and extrathyroid extension (Table 1, all P values > 0.05), lifestyle factors including smoking, alcohol consumption and fertility status (Table 2, all P values > 0.05), or for thyroid function parameters including TSH, fT3, fT4, TG and ATPO levels before or after surgery (Table 3, all P values > 0.05).

**Table 1:** Association between RET/PTC rearrangement and pathological parameters in thyroid cancer patients.

Variable	RET/PTC		*P*	RET/PTC1		*P*	RET/PTC3		*P*
	Negative	Positive		Negative	Positive		Negative	Positive	
**All thyroid cancer patients**									
Tumor Size (mean±SD, cm^2^)	2.53±4.09	2.93±2.81	0.64	2.53±4.07	2.95±2.88	0.637	2.55±3.77	6.23±7.82	0.181
T stage			0.345			0.414			0.470^a^
T1	26(83.9%)	5(16.1%)		26(83.9%)	5(16.1%)		30(96.8%)	1(3.2%)	
T2-4	62(75.6%)	20(24.4%)		63(76.8%)	19(23.2%)		81(98.8%)	1(1.2%)	
N stage			0.189 ^a^			0.093 ^a^			1.000^a^
N0	30 (85.7%)	5 (14.3%)		31 (88.6%)	4 (11.4%)		35 (100.0%)	0 (0.0%)	
N1	59 (74.7%)	20 (25.3%)		59 (74.7%)	20 (25.3%)		77 (97.5%)	2 (2.5%)	
Stage grouping			0.829			0.869			0.394 ^a^
I and II	66(78.6%)	19(21.4%)		66(78.6%)	18(21.4%)		82(97.6%)	2(2.4%)	
III and IV	23(76.7%)	7(23.3%0		24(80.0%)	6(20.0%)		30(100.0%)	0(0.0%)	
No. of lesions			0.069			0.207 ^a^			1.000 ^a^
Single	78 (81.2%)	18 (18.8%)		78 (81.2%)	18 (18.8%)		94 (97.9%)	2 (2.1%)	
Multiple	11 (61.1%)	7 (38.9%)		12 (66.7%)	6 (33.3%)		18 (100.0%)	0 (0.0%)	
Extrathyroid extension			0.417			0.227 ^a^			0.443^a^
No	64(76.2%)	20(23.8%)		64(76.2%)	20(23.8%)		83(98.8%)	1(1.2%)	
Yes	25(83.3%)	5(16.7%)		26(86.7%)	4(13.3%)		29(96.7%)	1(3.3%)	
**Thyroid cancer patients without nodular goiter or Hashimoto's thyroiditis**									
Tumor Size (mean±SD, cm^2^)	2.65±5.12	3.54±3.27	0.571	2.65±5.07	3.62±3.42	0.550	2.68±4.68	11.76±/	0.060
T stage			0.714 ^a^			1.000 ^a^			1.000 ^a^
T1	12(85.7%)	2(14.3%)		14(100.0%)	0(0.0%)		33(78.6%)	9(21.4%)	
T2-4	33(76.7%)	10(23.3%)		42(97.7%0	1(2.3%)		13(81.2%)	3(187.8%)	
N stage			1.000 ^a^			1.000 ^a^			1.000 ^a^
N0	10 (21.7%)	3 (25.0%)		11 (84.6%)	2 (15.4%)		13 (100.0%)	0 (0.0%)	
N1	36 (78.3%)	9 (75.0%)		36 (80.0%)	9 (20.0%)		44 (97.8%)	1 (2.0%)	
Stage grouping			0.710 ^a^			1.000 ^a^			0.939 ^a^
I and II	33(78.6%)	9(21.4%)		41(97.6%)	1(2.4%)		34(79.1%)	9(20.9%)	
III and IV	14(87.5%)	2(12.5%)		16(100.0%)	0(0.0%)		12(80.0%)	3(20.0%)	
No. of lesions			**0.018**			0.101			1.000 ^a^
Single	39 (86.7%)	6 (13.3%)		39 (86.7%)	6 (13.3%)		44 (97.8%)	1 (2.2%)	
Multiple	7 (53.8%)	6 (46.2%)		8 (61.5%)	5 (38.5%)		13 (100.0%)	0 (0.0%)	
Extrathyroid extension			1.000 ^a^			0.710 ^a^			0.259 ^a^
No	34(79.1%)	9(20.9%)		34(79.1%)	9(20.9%)		43(100.0%)	0(0.0%)	
Yes	12(80.0%)	3(20.0%)		13(86.7%)	2(13.3%)		14(93.3%)	1(6.7%)	

^a^, P values determined using Fisher’s exact test. Associations that reached statistical significance were highlighted in bold.

**Table 2:** Association between RET/PTC rearrangement and lifestyle of patients with thyroid cancer.

Variable	RET/PTC		*P*	RET/PTC1		*P*	RET/PTC3		*P*
	Negative	Positive		Negative	Positive		Negative	Positive	
**All thyroid cancer patients**									
Smoking			0.370 ^a^			0.362 ^a^			1.000 ^a^
No	84 (79.2%)	22 (20.8%)		85 (80.2%)	21 (19.8%)		104 (98.1%)	2 (1.9%)	
Yes	5 (62.5%)	3 (37.5%)		5 (62.5%)	3 (37.5%)		8 (100.0%)	0 (0.0%)	
Alcohol consumption			0.344 ^a^			0.342 ^a^			1.000 ^a^
No	82 (76.6%)	25 (23.4%)		83 (77.6%)	24 (22.4%)		105 (98.1%)	2 (1.9%)	
Yes	7 (100.0%)	0 (0.0%)		7 (100.0%)	0 (0.0%)		7 (100.0%)	0 (0.0%)	
Fertility condition ^b^			0.201			0.192			0.277 ^a^
No	11 (64.7%)	6 (35.3%)		11 (64.7%0	6 (35.3%)		16 (94.1%)	1 (5.9%)	
Yes	78 (80.4%)	19 (19.6%)		79 (81.4%)	18 (18.6%)		96 (99.0%)	1 (1.0%)	
**Thyroid cancer patients without nodular goiter or Hashimoto's thyroiditis**									
Smoking			0.273 ^a^			0.237 ^a^			1.000 ^a^
No	43 (81.1%)	10 (18.9%)		44 (83.0%)	9 (17.0%)		52 (98.1%)	5 (1.9%)	
Yes	3 (60.0%)	2 (40.0%)		3 (60.0%)	2 (40.0%)		5 (100.0%)	0 (0.0%)	
Alcohol consumption			0.573 ^a^			0.572 ^a^			1.000 ^a^
No	41 (77.4%)	12 (22.6%)		42 (79.2%)	11 (20.8%)		52 (98.1%)	1 (1.9%)	
Yes	5 (100.0%)	0 (0.0%)		5 (100.0%)	0 (0.0%)		5 (100.0%)	0 (0.%)	
Fertility condition ^b^			0.077 ^a^			0.056 ^a^			0.155 ^a^
No	5 (55.6%)	4 (44.4%)		5 (55.6%)	4 (44.4%)		8 (88.9%)	1 (11.1%)	
Yes	41 (83.7%)	8 (16.8%)		42 (85.7%)	7 (14.3%)		49 (100.0%)	0 (0.0%)	

^a^, P values determined using Fisher’s exact test

^b^, Fertility condition means females who have born children vs. females who do not have children.

**Table 3:** Association between RET/PTC rearrangements and thyroid function in all thyroid cancer patients.

Variable	RET/PTC				*P*^*a*^
	Negative		Positive		
	N	Median (25%-75%)	N	Median (25%-75%)	
**Pre-surgery**					
FT3	69	4.46 (4.18–4.78)	21	4.39 (3.94–4.80)	0.326
FT4	69	16.30 (15.07–17.93)	21	17.14 (14.83–18.61)	0.633
TSH	69	2.25 (1.30–3.19)	21	2.05 (1.60–3.16)	0.909
ATPO	69	12.50 (6.47–25.04)	21	12.69 (7.07–40.85)	0.596
TG	69	19.67 (10.65–53.81)	21	19.81 (4.45–62.94)	0.928
**One month post-surgery**					
FT3	69	4.97 (4.17–5.66)	21	4.87 (3.47–5.81)	0.664
FT4	69	21.12 (18.03–24.31)	21	20.75 (16.24–26.41)	0.557
TSH	69	0.60 (0.30–1.66)	21	0.80 (0.23–7.52)	0.675
ATPO	69	12.56 (6.35–23.59)	21	13.52 (8.53–22.63)	0.367
TG	69	2.21 (0.40–5.58)	21	0.93 (0.13–5.29)	0.403

^a^, Analysis performed using the Mann-Whitney test.

### Influence of concomitant diseases on RET/PTC rearrangement in thyroid cancer

We further examined the effect of concomitant diseases on the association between RET/PTC rearrangement and clinicopathologic characteristics, lifestyle and thyroid function in PTC. Of the 114 PTC cases, 58 (50.8%) cases did not have concomitant disease, while the remaining 56 cases (49.12%) demonstrated concomitant disease including 38 cases of nodular goiter, 15 cases of Hashimoto's thyroiditis and three cases with a combination of nodular goiter and Hashimoto's thyroiditis. The subjects were divided into two groups: PTC with concomitant disease and PTC without concomitant disease.

For the subgroup of PTC with concomitant disease, similar results were observed as those in the total PTC cases when concomitant disease was not considered. No association was found between RET/PTC rearrangements and clinicopathologic features, lifestyle or thyroid function (data not shown).

For the subgroup of PTC patients without concomitant disease, RET/PTC rearrangement showed a statistically significant association with the number of lesions in thyroid gland (Table 1, P = 0.018). By using a likelihood-ratio regression analysis, the RET/PTC-positive PTC cases showed an increased risk of multifocal cancer in the thyroid gland (OR = 5.57, 95% CI, 1.39–22.33). With respect to thyroid function, serum TSH levels measured one month after surgery were found to be higher in RET/PTC-positive PTC cases as compared to RET/PTC-negative PTC cases (P = 0.037, Table 4). Changes in TSH levels before and after surgery were more significant for RET/PTC-positive patients than patients with no rearrangement (P = 0.003, Table 5). No difference in fT3, fT4, TG and ATPO levels were found between RET/PTC positive and negative cases either before or after thyroidectomy (Tables 4 and 5).

**Table 4:** Association between RET/PTC rearrangement and thyroid function in thyroid cancer patients without nodular goiter or Hashimoto's thyroiditis.

Variable	RET/PTC				*P*^*a*^
	Negative		Positive		
	N	Median (25%-75%)	N	Median (25%-75%)	
**Pre-surgery**					
FT3	34	4.49 (4.16–4.77)	11	4.39 (4.14–4.92)	0.402
FT4	34	16.62 (14.78–18.27)	11	17.95 (14.44–18.88)	0.261
TSH	34	1.91 (1.21–2.76)	11	2.06 (1.60–3.30)	0.899
ATPO	34	6.03 (12.50–18.95)	11	8.47 (6.89–20.35)	0.402
TG	34	19.75 (12.65–34.10)	11	36.22 (14.13–68.05)	0.894
**One month post-surgery**					
FT3	34	4.85 (4.17–5.65)	11	4.43 (1.33–5.62)	0.144
FT4	34	20.28 (17.79–23.21)	11	17.60 (3.71–21.10)	0.074
TSH	34	0.62 (0.33–1.43)	11	1.90 (0.38–100.00)	**0.037**
ATPO	34	11.47 (6.21–15.85)	11	11.15 (7.80–19.22)	0.558
TG	34	3.03 (1.11–5.64)	11	0.93 (0.12–8.20)	0.611

^a^, Analysis was performed using the Mann-Whitney test.

**Table 5:** Association between RET/PTC rearrangements and changes in thyroid function in thyroid cancer patients.

Variable	RET/PTC				*P*^*a*^
	Negative		Positive		
	N	Mean	N	Mean	
**All thyroid cancer patients**					
ΔFT3 _(pre-post)_	69	-0.51	21	-0.25	0.602
ΔFT4 _(pre-post)_	69	-4.99	21	-2.95	0.323
ΔTSH _(pre-post)_	69	-1.96	21	-15.63	0.098
ΔATPO _(pre-post)_	69	12.96	21	19.35	0.519
ΔTG _(pre-post)_	69	68.56	21	36.92	0.427
**Thyroid cancer patients without nodular goiter or Hashimoto's thyroiditis**					
ΔFT3 _(pre-post)_	34	-0.47	11	0.47	0.111
ΔFT4 _(pre-post)_	34	-4.10	11	1.14	0.129
ΔTSH _(pre-post)_	34	-0.88	11	-26.69	**0.003**
ΔATPO _(pre-post)_	34	2.47	11	2.40	0.981
ΔTG _(pre-post)_	34	38.48	11	35.01	0.910

^a^ Analysis was performed using a Student’s t-test.

### Risk factor for concomitant thyroid benign diseases in PTC patients

Since associations between RET/PTC and multifocal lesions was merely observed in PTC without concomitant thyroid benign disease as described above, we further explored possible risk factor affecting the development of concomitant diseases in PTC. In univariate analysis, no association was observed between RET/PTC and status of concomitant diseases in PTC (S2 Table). In multivariate analysis, we found female gender and multifocal cancers in thyroid gland were positive associated with the occurrence of concomitant thyroid benign diseases in PTC patients (OR = 2.79 and 4.31, respectively, S3 Table).

## Discussion

The objective of this study was to examine the influence of RET/PTC rearrangements on thyroid hormone homeostasis, and to determine whether concomitant diseases like nodular goiter and Hashimoto's thyroiditis had an effect on relationship between RET/PTC rearrangement and PTC. In the subpopulation of PTC patients without concomitant disease, subjects with RET/PTC rearrangements showed an increased risk of developing multifocal cancer in the thyroid gland, and demonstrated higher TSH levels at one month following surgery.

Multifocality is a potential prognostic factor in thyroid cancer. In this study, RET/PTC-positive patients showed an increased risk of developing multifocal thyroid cancers. Two different theories regarding the origin of multiple foci have been proposed. One theory is that multiple foci are clonal in origin and arise due to the intraglandular metastasis of a single tumor, which may indicate an increase in the aggressiveness of the cancer [7, 8]. Alternatively, multiple foci may arise independently, which could explain contralateral lobe recurrences years after initial therapy [4, 5]. Regardless of whether multifocality arises *de novo* or from a single clone, tumor multifocality has been reported to be associated with local and distant metastases and tumor recurrence [6, 9].

In this study, we demonstrated that an association between RET/PTC and multifocal thyroid cancer existed only in the subgroup of PTC patients without concomitant disease. Rhoden and colleagues reported that follicular cells of Hashimoto’s thyroiditis share low-level RET/PTC expression with papillary carcinoma, suggesting that RET/PTC expression did not predict the development of PTC in patients with thyroiditis [2]. In this study, a large proportion of PTC cases were accompanied by inflammatory disease (49.12%), which could have a confounding effect on the correlation between RET/PET rearrangements the manifestations of PTC. Removal of the confounding factor permits a more accurate determination of correlation. Our study suggested that if RET/PTC rearrangement was detected in PTC cases without concomitant disease, a more aggressive treatment approach could be required.

Among the common thyroid hormones, we found that RET/PTC rearrangements were associated with higher serum TSH levels post-surgery in the PTC patients without concomitant disease, suggesting that surgery alone may not be sufficient to normalize thyroid hormone levels in PTC patients carrying the RET/PTC fusion gene. TSH is a sensitive determinant of thyroid dysfunction, and is recommended to serve as an indicative index in the initial evaluation of patients presenting with thyroid nodules as well as a surveillance indicator after thyroidectomy [9]. It is documented that TSH has a trophic effect on thyroid cancer growth, likely mediated by TSH receptors on tumor cells, and decreased serum TSH concentrations at presentation are an independent predictor of thyroid malignancy [7]. Sugg SL and his colleagues reported that TSH action could be disrupted by RET/PTC at several points [8]. Elevated postoperative serum TSH levels could be explained at least in part by removal of tumor tissue carrying the RET/PTC fusion gene, which may confer an increased risk of tumor recurrence and metastasis. The mechanisms through which RET/PTC rearrangements in thyroid carcinoma affect serum TSH levels remain to be established. Further research is required to determine how RET/PTC rearrangements affect levels of thyroid hormones such as TSH.

Several limitations should also be noted. First, the majority of PTC patients enrolled in the current study did not demonstrate distant metastases (only two cases occur metastases); therefore, we could not establish an association between RET/PTC rearrangement and distant metastases. Second, we couldn’t include the factor of PTC variants into analysis because the “type of PTC variants” is not reported in pathological diagnosis routinely in our cancer center, which should be assessed in further studies.

In conclusion, this study building upon previous work examining the relationship between RET/PTC rearrangements and thyroid cancer. We found that serum TSH in RET/PTC-positive thyroid cancer patients without nodular goiter or Hashimoto's thyroiditis increased following surgery. This likely creates a new homeostatic state, in which cells RET/PTC rearrangements may interfere with the TSH signaling pathway.

## Supporting Information

S1 Table The sequence of primers and probes for RT-PCR. (DOCX)

S2 Table Association between RET/PTC and status of concomitant diseases of nodular goiter and Hashimoto's thyroiditis in PTC. (DOCX)

S3 Table Multivariate analysis for the status of concomitant diseases of nodular goiter and Hashimoto's thyroiditis in PTC. (DOCX)
