# Supplementary material for: RET/PTC Rearrangements Are Associated with Elevated Postoperative TSH Levels and Multifocal Lesions in Papillary Thyroid Cancer without Concomitant Thyroid Benign Disease
Source: PLoS One. 2016 Nov 1;11(11):e0165596. doi: 10.1371/journal.pone.0165596 (PMC5089556; doi:10.1371/journal.pone.0165596)
Supplement: S1 Table — (DOCX) [file pone.0165596.s001.docx]

**S1 Table The sequence of primers and probes for RT-PCR**

| Rearrangement | Primer and probe | Sequence (5'-3') |
| --- | --- | --- |
| RET/PTC1 | Forward primer | GCCAGCGTGACCATCGAG |
|  | Reverse primer | GCCGTTGCCTTGACCACT |
|  | Probe | FAM-CCTCGGAAGAACTTGGT-MGB |
| RET/PTC2 | Forward primer | GACAGCTATAGAAGAATCCTCATGGA |
|  | Reverse primer | GCCGTTGCCTTGACCACT |
|  | Probe | FAM-CCTCGGAAGAACTTGGT-MGB |
| RET/PTC3 | Forward primer | CCTTGGAGAACAGTCAGGAGGA |
|  | Reverse primer | AGTGGTCAAGGCAACGGC |
|  | Probe | FAM-CCTCGGAAGAACTTGGT-MGB |
