# Supplementary material for: RET/PTC Rearrangements Are Associated with Elevated Postoperative TSH Levels and Multifocal Lesions in Papillary Thyroid Cancer without Concomitant Thyroid Benign Disease
Source: PLoS One. 2016 Nov 1;11(11):e0165596. doi: 10.1371/journal.pone.0165596 (PMC5089556; doi:10.1371/journal.pone.0165596)
Supplement: S2 Table — (DOCX) [file pone.0165596.s002.docx]

**S2 Table** **Association between RET/PTC and status of concomitant diseases of nodular goiter and Hashimoto's thyroiditis in PTC**

| Variable | Status of concomitant disease in PTC | | P |
| --- | --- | --- | --- |
|  | No | Yes |  |
| RET/PTC |  |  | 0.745 |
| Negative | 46(79.3%) | 43(76.8%) |  |
| Positive | 12(20.7%) | 13(23.2%) |  |
|  |  |  |  |
| RET/PTC1 |  |  | 0.578 |
| Negative | 47(81.0%) | 43(76.8%) |  |
| Positive | 11(19.0%) | 13(23.2%) |  |
| RET/PTC3 |  |  |  |
| Negative | 57(98.3%) | 55(98.2%) | 1.000^a^ |
| Positive | 1(1.7%) | 1(1.8%) |  |

^a^, P values determined using Fisher’s exact test.
