# Supplementary material for: RET/PTC Rearrangements Are Associated with Elevated Postoperative TSH Levels and Multifocal Lesions in Papillary Thyroid Cancer without Concomitant Thyroid Benign Disease
Source: PLoS One. 2016 Nov 1;11(11):e0165596. doi: 10.1371/journal.pone.0165596 (PMC5089556; doi:10.1371/journal.pone.0165596)
Supplement: S3 Table — (DOCX) [file pone.0165596.s003.docx]

**S3 Table** **Multivariate analysis for the status of concomitant diseases of nodular goiter and Hashimoto's thyroiditis in PTC**

| Factor | OR(95%CI) | P^a^ |
| --- | --- | --- |
| Sex (female=1, male=0) | **2.79(1.04-7.52)** | **0.042** |
| Age (yrs) | 0.98(0.94-1.02) | 0.274 |
| Tumor size | 1.04(0.94-1.17) | 0.482 |
| Stage grouping (III+IV =1, I+II=0) | 1.60(0.50-5.18) | 0.432 |
| No. of lesion (Multiple=1, single=0) | **4.31(1.22-15.16)** | **0.023** |
| Extrathyroid extension (Yes=1, No=0) | 1.25(0.31-2.08) | 0.644 |
| RET/PTC (Yes=1, No=0) | 0.44(0.15-1.24) | 0.121 |

^a^, Analysis was performed by multifactorial logistic regression analysis using likelihood-ratio test.
